# Bifunctional V-doped NiCoP nanowires for high-efficiency electrolysis

**DOI:** 10.1039/d5ra09888b

**Published:** 2026-03-17

**Authors:** Yongli Tong, Xuan Zhao, Yu Dong, Ende Wang

**Affiliations:** a School of Science, Shenyang Ligong University Shenyang 110159 China tyl.tongyongli@163.com; b School of Materials Science and Engineering, Shenyang University of Technology Shenyang 110870 China ende_wang@163.com

## Abstract

The development of highly efficient and economically viable bifunctional electrocatalysts is essential for overall water splitting in alkaline environments. Therefore, we synthesize V-doped NiCoP one-dimensional nanowire catalysts using hydrothermal and chemical vapor deposition methods. The as-obtained NCP-2 sample exhibits superior electrocatalytic performance, which might be attributed to the moderate vanadium doping that increases the density of electrochemically active sites and modulates the electronic structure of NiCoP. The NCP-2 sample shows an overpotential of 74.3 mV at 10 mA cm^−2^ with a small Tafel slope of 83.8 mV dec^−1^ during HER activity. In the OER process, NCP-2 exhibits an overpotential of 280 mV at 20 mA cm^−2^ (79.2 mV dec^−1^). Meanwhile, the NCP-2 nanowire arrays possess a low cell voltage of 1.55 V at 10 mA cm^−2^. This material shows significant potential for applications in sustainable energy systems.

## Abstract 2

The development of highly efficient and economically viable bifunctional electrocatalysts is essential for overall water splitting in alkaline environments.

## Graphical abstract

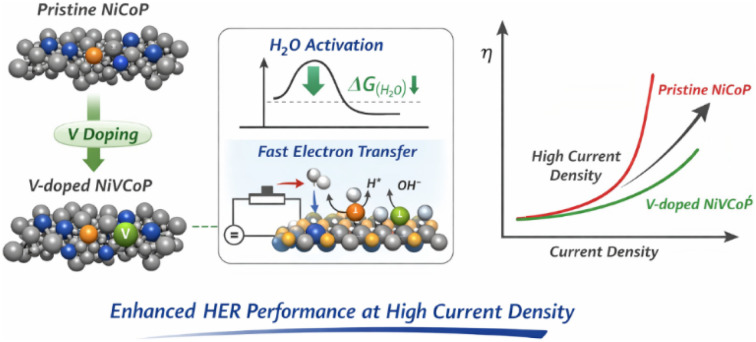

## 1. Introduction

Hydrogen is widely regarded as a clean and sustainable energy and represents a promising substitute for conventional fossil fuels in addressing the escalating energy shortage and environmental contamination.^1–3^ Among the available strategies for hydrogen generation, electrocatalytic water splitting has attracted extensive attention owing to its potential for large-scale hydrogen production with negligible carbon emissions. Achieving efficient water electrolysis strongly depends on advanced electrocatalysts, which can effectively promote reaction kinetics and minimize overpotentials associated with both the hydrogen evolution reaction (HER) and oxygen evolution reaction (OER).^4–6^ At present, noble metal-based catalysts, such as Pt/C for HER and IrO_2_/RuO_2_ for OER, exhibit outstanding catalytic performance; however, their limited availability and high cost greatly hinder practical and large-scale deployment. In addition, most HER catalysts show optimal activity in acidic environments, whereas OER typically proceeds more favorably under alkaline conditions, making it difficult to construct bifunctional catalysts capable of driving both reactions in a single electrolyte. Consequently, the exploration of noble-metal-free, highly efficient, and durable bifunctional electrocatalysts is of critical importance for sustainable overall water splitting.

A variety of electrocatalysts based on abundant and inexpensive elements have emerged as viable candidates to replace noble-metal catalysts, including transition metal oxides,^7^ metal sulfides,^8^ and metal phosphides.^9^ Among these candidates, NiCoP, a representative transition metal phosphide (TMP), has drawn considerable interest due to its metallic characteristics and the strong synergistic interaction between Ni and Co.^10–12^ NiCoP can undergo surface reconstruction to generate metal oxyhydroxide (M–OOH) species that are highly active toward the OER, while the metal–phosphorus (M–P) bonds facilitate hydride and proton adsorption, thereby enhancing HER performance.^13–15^ Despite these advantages, its practical catalytic efficiency remains limited by insufficient electrical conductivity and a scarcity of accessible active sites, which impede its application in efficient overall water splitting.

In general, the electrocatalytic activity of a catalyst is governed by number of accessible active sites and the inherent activity of each site. Doping strategies and structural morphology design are both effective approaches to enhance catalytic performance. Introducing metal cations as dopants can induce lattice distortion and modulate the electronic structure of catalysts, thereby increasing the density of exposed active sites, optimizing adsorption behavior, and ultimately enhancing electrocatalytic activity.^16,17^ For example, Kim *et al.*^18^ reported the fabrication of Ru-doped NiCoP nanoparticles *via* a one-step synthesis strategy, which demonstrated outstanding OER activity with an overpotential of only 281 mV to reach 10 mA cm^−2^. The introduction of Ru atoms generated a high density of Ni and Co vacancies within the NiCoP lattice, resulting in a more defect-rich structure and a large number of active sites compared to the pristine NiCoP counterpart. Zhang's group^19^ constructed two-dimensional vanadium-doped NiCoP nanosheets, which exhibited excellent HER performance. At a current density of 100 mA cm^−2^, the overpotential reached 332 mV.

Herein, we synthesize V-doped NiCoP nanowires using hydrothermal and chemical vapor deposition methods. Vanadium is particularly well suited as a dopant for vacancy engineering in TMPs, owing to its ability to adopt multiple high oxidation states (V^3+^/V^4+^/V^5+^) in phosphide-based materials.^20^ Moreover, under alkaline conditions, V species tend to form thermodynamically unstable and partially soluble vanadium-containing intermediate, which can promote the generation of structural defects and increase the number of catalytically active sites during electrochemical operation. Therefore, the incorporation of V dopants during TMP synthesis can effectively modulate both the electronic structure and crystal lattice, creating abundant vacancies and active site.^21^ The NiCoP-2 sample shows excellent electrocatalytic performance. The overpotential of NCP-2 sample is 74.3 mV at 10 mA cm^−2^ during HER process; in the oxygen evolution reaction (OER), NCP-2 product exhibits an overpotential of 280 mV at 20 mA cm^−2^.

## 2. Experimental section

### 2.1. Materials

All materials were used as received, without further purification. Cobalt nitrate (Co(NO_3_)_2_·6H_2_O), nickel nitrate hexahydrate (Ni(NO_3_)_2_·6H_2_O) and sodium vanadate (Na_2_VO_3_·6H_2_O) were sourced from Tianjin Damao Chemical Reagent Factory in Tianjin, China. Ethanol (AR, 99.5%), ammonium fluoride (NH_4_F), potassium hydroxide (KOH) and urea were supplied by Sinopharm Chemical Reagent Company in Shanghai, China. NF of 1 mm thick was procured from Kunshan Guangshengjia New Material Co. in Kunshan, China. Deionized water (DI) was produced using a homemade ultrapure water system.

### 2.2. Preparation of V-NiCoP catalysts

First, the nickel foam was cleaned by alternately sonicating with deionized water and alcohol three times, each for 30 minutes. It was then cut into a 3 cm × 4 cm piece for later use. 1 mmol of Ni(NO_3_)_2_·6H_2_O, 1 mmol of Co(NO_3_)_2_·6H_2_O, 5 mmol of NH_4_F, and 3 mmol of urea were dissolved in 60 ml of DI water and stirred until the solution became clear. Four additional solutions were prepared using the same ratios, and sodium metavanadate (NaVO_3_) was added to each solution in amounts of 0.05, 0.1 and 0.2 mmol, respectively. The mixed solutions were transferred into a 100 ml reaction vessel and sealed. They were then heated to 120 °C in an oven and maintained at that temperature for 6 h. Then, the samples were repeatedly washed with DI water and alcohol to remove surface impurities. Finally, they were dried in a 60 °C oven. The obtained samples were further phosphidized using a tubular furnace though a chemical vapor deposition method. At the center of the tube, the samples and 1 g of sodium dihydrogen phosphate were placed in boats, respectively. The tubular furnace was heated to 350 °C at a rate of 2 °C min^−1^ and held for 2 h. The V-doped NiCoP samples have been successfully prepared. Based on the increasing vanadium content, the resulting samples were labeled as NCP, NCP-1, NCP-2, NCP-3.

### 2.3. Structural characterization

The materials' structure and morphology were analyzed using X-ray photoelectron spectroscopy (Thermo Scientific Kα with Al Kα source, Thermo Fisher Scientific, Waltham, MA, USA) and X-ray diffraction (Cu Kα radiation, Ultima IV, 40 kV, Rigaku Corporation, Tokyo, Japan). Their morphology was examined with a scanning electron microscope (Gemini 300-71-31, Carl Zeiss AG, Oberkochen, Germany).

### 2.4. Electrochemical characterization

All electrochemical tests were conducted using an electrochemical workstation (Shanghai Chenhua CHI 660e, Shanghai, China) with a 1 M KOH solution as the electrolyte. A Hg/HgO electrode served as the reference electrode. Graphite rods and platinum sheets were employed as counter electrodes for the hydrogen evolution reaction (HER) and oxygen evolution reaction (OER), respectively, while the synthesized samples acted as working electrodes. The potential values were derived by referencing the reversible hydrogen electrode (RHE) using the following equation:

1 *E*_RHE_ = *E* (Hg/HgO) + 0.098 + 0.591 × pH (∼13.7)

## 3. Results and discussion

### 3.1. Phase and structural analyses

**Fig. 1:**
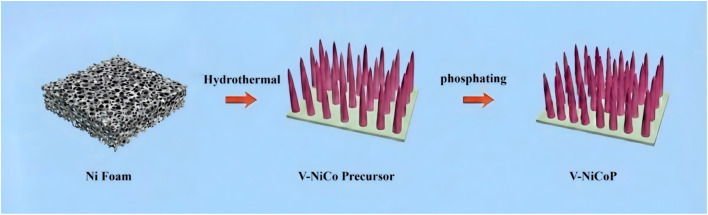
Synthesis schematic of V-doped NiCoP nanowire on nickel foam.

Schematic illustration is shown for the formation procedure of V doped NiCoP nanowire arrays on nickel foam (NF) in Fig. 1. Briefly, cobalt, nickel, and vanadium salts are mixed in a defined molar ratio and grown on NF *via* a hydrothermal process to obtain the V-doped NiCo precursor. This precursor is then subjected to a phosphidation treatment in a tube furnace, yielding the V-doped NiCoP material. The possible reactions involved in the phosphidation step are as follows:2
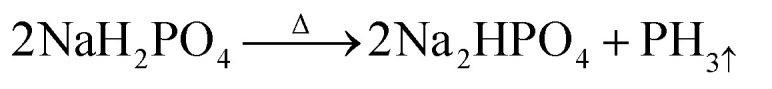

3 Co^2+^ + 2PH_3_ → Co–P + 6H^+^ + P↓

4 Ni^2+^ + 2PH_3_ → Ni–P + 6H^+^ + P↓

**Fig. 2:**
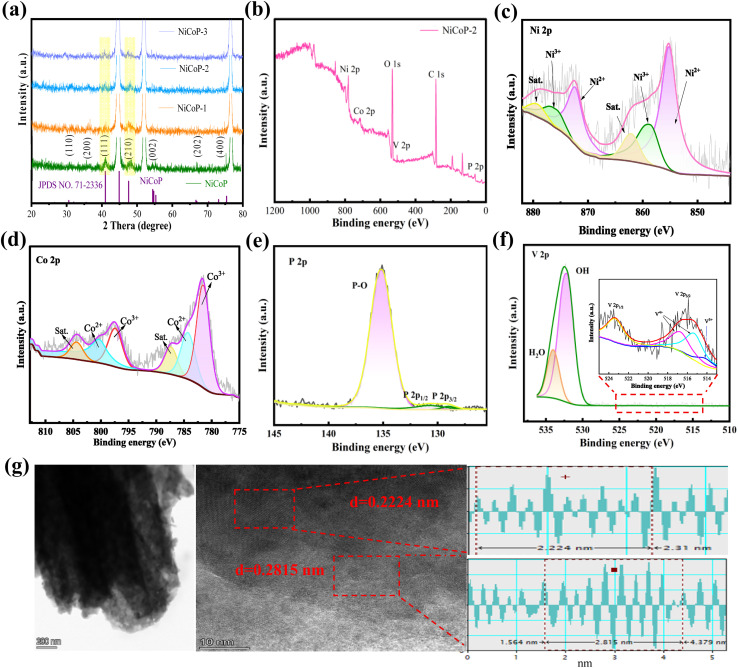
Structural characterization (a) XRD patterns (b) XPS patterns of survey spectra (c) Ni 2p (d) Co 2p (e) P 2p (f) V 2p (g) HRTEM of NiCoP-2.

XRD is used to describe the structure and phase composition of the prepared sample in Fig. 2a. Peaks at 44.6, 51.9 and 76.5° correspond to characteristic peaks of nickel foam (JCPDS no. 04-0850). The peaks at 30.6°, 35.5°, 40.9°, 47.6°, 54.7°, 66.7° and 75.2° are consistent with (110), (200), (111), (210), (002), (202) and (400) crystal planes NiCoP (JCPDS: 71-2336), respectively. These diffraction peaks at 24.8°, 35.6°, 38.8°, 48.4°, 58.5°, 61.4°, 67.1°, 67.6°, and 78.4° are assigned to the (−111), (200), (012), (022), (131), (−313), (113), (311), and (042) reflections of CoP_2_ in Fig. S1, respectively, which are in good agreement with the standard JCPDS card (No. 77-0263). With the vanadium content increasing from 0.05 mmol to 0.2 mmol, the (111) and (210) crystal planes of NiCoP shift gradually to the left and no other new diffraction peaks are produced. Upon vanadium doping, the diffraction peaks shift to lower angles, evidencing successful incorporation and lattice expansion. This geometric distortion, driven by the disparate atomic dimensions and electronic properties of V relative to Ni/Co, creates localized strain fields that modify the d-band electronic structure of metal sites, potentially optimizing their catalytic activity through altered adsorbate binding strengths.

XPS is carried out to further determine the composition and electronic structure of the material. The survey spectrum shows the presence of Ni, Co, P, V, C and O elements in Fig. 2b. Among these, Ni, Co, P, and V are derived from the prepared sample V-NiCoP. Fig. S2 shows the atomic percentage of each element, with C, O, P, Co, Ni, and V accounting for 60.7%, 25%, 11%, 2.5%, 0.7% and 0.2%, respectively. Ni 2p spectra (Fig. 2c) reveal four prominent peaks.^22,23^ The binding energy at 855.3 eV is associated with Ni 2p_3/2_ peaks, while the peaks at 872.6 eV correspond to Ni 2p_1/2_ peaks. Additionally, there are two satellite peaks at 859.5 and 877.8 eV, indicative of higher oxidation states. The peaks at 855.6 and 873.9 eV are assigned to Ni^2+^, whereas the peaks at 857.6 and 875.8 eV are linked to Ni^3+^. Compared to the Ni 2p peak in pristine NiCoP, the binding energy exhibits a negative shift of approximately 0.3 eV.^24^ This phenomenon arises because nickel possesses greater electronegativity than vanadium, facilitating electron transfer from vanadium to nickel. The resulting increase in electron density and the lowering of the oxidation state leads to a decrease in binding energy.^25^ The Co 2p XPS spectra (Fig. 2d) exhibit a spin–orbit doublet with Co 2p_3/2_ and Co 2p_1/2_ components centered at 781.7 eV and 797.6 eV, respectively, yielding a spin–orbit splitting energy of ∼15.9 eV, consistent with reported values for cobalt phosphides.^26,27^ The binding energies at 781.5 and 797.5 eV are ascribed to Co^3+^, while those at 784.1 and 799.9 eV are linked to Co^2+^. Likewise, given that cobalt is more electronegative than vanadium, it accepts electrons from vanadium, leading to a decreased oxidation state and enhanced electron density, with the binding energy shifting negatively by approximately 0.3 eV as well.^24,25^Fig. 2e shows two peaks of P 2p_1/2_ and P 2p_3/2_, located at 129.5 and 130.5 eV, respectively. The peak at 135.3 eV results from the oxidation of phosphide by the superficial oxidation of metal phosphide due to the direct air contact of the sample.^28^ From Fig. 2f, the peak at 530.3 eV can be attributed to the V–O bonding, which is a consequence of the oxidation of vanadium.^29^ The V 2p XPS spectrum confirms vanadium exists in mixed oxidation states. The V 2p envelope is deconvoluted into three components at 514.2 eV (V^3+^), 515.3 eV and 516.7 eV (V^4+^), with the corresponding V 2p peak at 523.35 eV.^20^ Notably, the 516.7 eV binding energy aligns with reported values for V^4+^. This V^3+^/V^4+^ redox couple endows NiCoP with dynamic electronic flexibility, enabling adaptive modulation of active metal centers under varying electrochemical conditions.^21^ This multivalent-induced charge redistribution optimizes hydrogen adsorption/desorption during HER and stabilizes high-valence Ni/Co active species for OER, thereby delivering enhanced bifunctional electrocatalytic performance. The HRTEM image in Fig. 2g reveals lattice fringes with spacings of 0.2224 and 0.2815 nm, which can be indexed to the (111) and (101) planes of NiCoP, respectively, confirming the successful formation of the NiCoP structure. Notably, both values exceed the corresponding standard spacings (0.2200 and 0.2793 nm), indicating lattice expansion associated with the incorporation of vanadium into the NiCoP framework. Lattice expansion and local lattice disorder, together with XPS peak shifts may collectively indicate the generation of defects.

**Fig. 3:**
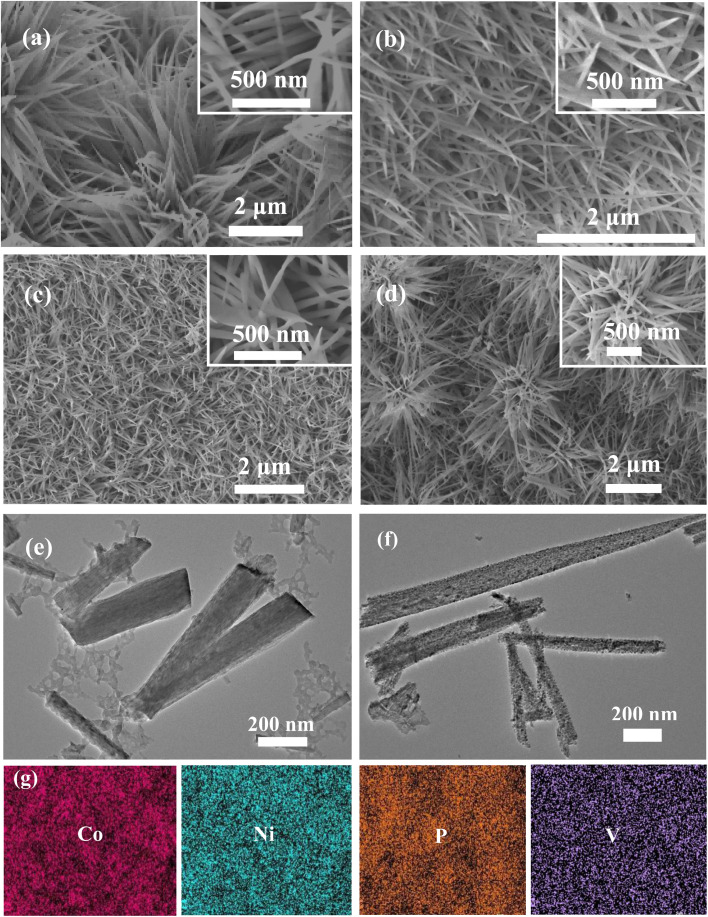
SEM images (a) NCP sample (b) NCP-1 sample (c) NCP-2 sample (d) NCP-3 sample. TEM image of (e) V doped NiCo-precursor (f) NCP-2 sample (g) elemental mapping of NiCoP-2.

SEM is employed to examine the surface structure of the prepared samples. Fig. 3a shows the morphology of NiCoP sample, where nanowires are uniformly grown on the surface of nickel foam. The nanowires show a diameter of approximately 10–20 nm and a length of several micrometers. Fig. 3b–d display the morphologies of NCP-1, NCP-2 and NCP-3. It is evident that they all retain the nanowire structure, with no significant morphological changes observed due to the doping of vanadium. However, a closer examination reveals that as the vanadium content increases, the diameter of the nanowires slightly thickens. Vanadium doping may influence the growth mechanism of the nanowires, leading to the enlargement or aggregation of the crystalline grains. Moreover, TEM tests are conducted on the morphology before and after phosphidation to further observe the morphological changes. Fig. 3e shows the morphology of the V-NiCo precursor before phosphidation, where the nanowires appear smooth. After phosphidation, the V doped NiCoP-2 nanowires become uneven (Fig. 3f), with a significantly increased surface area, indicating enhanced catalytic performance. As shown in Fig. 3g, elemental mapping reveals a homogeneous distribution of Co, Ni, P, V elements within the NiCoP sample.

### 3.2. Electrochemical testing of HER

**Fig. 4:**
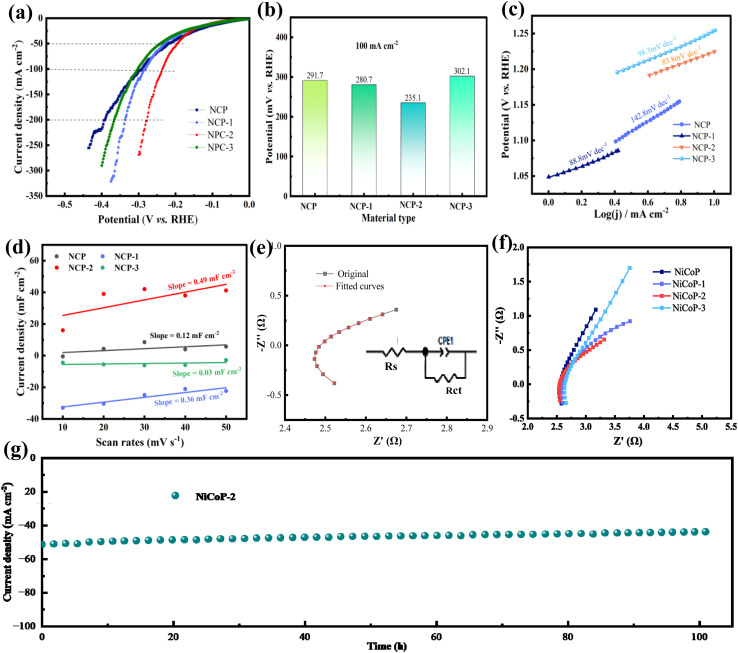
HER performance of the as-fabricated products (a) LSV curves (b) overpotential values at 100 mA cm^−2^ (c) Tafel curves (d) double-layer capacitance (e) Nyquist comparison plot of original and fitted curves (f) Nyquist plots (g) chronopotentiometric tests.

To assess the practical applicability of the synthesized samples, HER performances are investigated in a three-electrode system using a 1 M KOH electrolyte. The IR-compensated LSV curves are carried out in Fig. 4a. NCP-2 demonstrates excellent HER activity, requiring only 74.2 mV of overpotential to reach a current density of 10 mA cm^−2^. As the current density increases to 50 mA cm^−2^, the overpotentials rise to 189.2 mV. It reaches 234.1 mV at 100 mA cm^−2^, which is significantly lower than those of NCP (291.7 mV), NCP-1 (280.7 mV), and NCP-3 (302.1 mV). It clearly illustrates the superior catalytic performance of NCP-2. The bar chart in Fig. 4b shows the overpotential of various samples at a current density of 100 mA cm^−2^, providing a more intuitive comparison. The overpotential of NCP-2 is significantly lower than that of the other samples. At low current densities, all samples can readily complete the Volmer–Heyrovsky steps, resulting in negligible differences in overpotential. As the current density increases, materials without doping or with suboptimal doping levels experience limitations in electron transport or H* coverage, leading to a rapid rise in overpotential. In contrast, NCP-2, with an optimal doping level, exhibits enhanced electronic conductivity, moderate H* coverage, and a reduced water dissociation energy barrier, which together slow the increase in overpotential. Consequently, the superiority of NCP-2 becomes more pronounced at high current densities. Table 1 demonstrates that the overpotential of NCP-2 at 10 mA cm^−2^ outperforms that reported in several previous studies.^30–34^

**Table 1:** A comparison for HER with previous reported Ni, Co-based materials

Electrocatalysts	Overpotential (10 mA cm^−2^)	Electrolyte	References
NiCoS	303 mV	1 M KOH	30
NiCo_2_O_*x*_	164 mV	1 M KOH	31
Co_5_MoP	173 mV	1 M KOH	32
NiFeP@C	160 mV	1 M KOH	33
NiFeP@NiP@NF	227 mV	1 M KOH	34
NiCoP-2	74.3 mV	1 M KOH	This work

The HER reaction in an alkaline solution involves the following two processes:^35,36^H_2_O + e^−^ → H_ads_ + OH^−^ (Volmer reaction)

5 H_2_O + H_ads_ + e^−^ → H↑ + OH^−^ (Heyrovsky reaction)

H_2_O + e^−^ → H_ads_ + OH^−^ (Volmer reaction)

6 H_ads_ + H_ads_ → H↑ (Tafel reaction)

The intrinsic activity of HER can be characterized by the Tafel slope in Fig. 4c. Among all the samples, NCP-2 shows the lowest Tafel slope of 83.8 mV dec^−1^, which is lower than the NCP sample (142.8 mV dec^−1^), NCP-1 (88.8 mV dec^−1^), and NCP-3 (98.3 mV dec^−1^). It indicates that the HER follows a Volmer–Heyrovsky mechanism with mixed kinetic control. The incorporation of V modulates the electronic structure of NiCoP, facilitating water dissociation and optimizing H* adsorption, thereby accelerating the Volmer step while enabling the Heyrovsky step to participate in the rate-determining process.

To assess the inherent activity of catalysts, the electrochemical active surface area (ECSA) is evaluated through the electrochemical double-layer capacitance method (*C*_dl_). The *C*_dl_ is measured using a straightforward cyclic voltammetry (CV) technique at varying scan rates (10, 20, 30, 40, 50 mV s^−1^) within the potential range of 0.67–0.87 V (*vs.* RHE), with no faradaic current features observed. Fig. 4d shows NCP-2 sample possesses the ECSA of 0.49 mF cm^−2^, meaning a high specific surface area with abundant active sites. It can significantly enhance the electrochemical process. EIS characterization is employed to analyze the charge transfer kinetics. The EIS measurements are fitted using an equivalent circuit model, where the solution resistance (*R*_s_) is connected in series with a parallel arrangement of the charge-transfer resistance (*R*_ct_) and a constant phase element (CPE). The CPE accounts for the non-ideal behavior of the double-layer capacitance at the electrode/electrolyte interface. The equivalent circuit is illustrated in Fig. 4e. The curve fitted with the equivalent circuit model closely coincides with the experimental data, demonstrating the reliability of the chosen model. The fitting results indicate that the equivalent resistance of the NiCoP-2 sample is 2.5 Ω. Fig. 4f presents a comparison of the impedance spectra of samples with different vanadium doping contents. The NiCoP-2 sample exhibits the smallest semicircle radius, indicating the lowest charge-transfer resistance (*R*_ct_), the fastest HER kinetics, and the best catalytic performance. With increasing vanadium content, the semicircle radius first decreases and then increases. This suggests that an appropriate amount of vanadium doping can reduce the charge-transfer resistance, whereas excessive vanadium doping hinders charge-transfer pathways and leads to increased resistance. The long-cycle performance of the NCP-2 material is tested to explore its stability and durability. As shown in the Fig. 4g, there is no significant decrease in current density after 100 h of cycling. The excellent cycling stability stems from the synergistic stabilization of the chemically bonded interface, V-doping, and 1D nanostructure. The *in situ* grown nanowires prevent delamination and dissipate mechanical stress from gas bubble evolution, while V-doping strengthens the lattice, suppresses phosphorus leaching, and inhibits metal dissolution *via* charge compensation. Furthermore, surface reconstruction forms a stabilized core–shell architecture where V-anchored (oxy)hydroxide active layers protect the conductive phosphide core, and the 3D Ni foam backbone ensures persistent electron transport throughout long-term redox cycling.

### 3.3. Electrochemical testing of OER

**Fig. 5:**
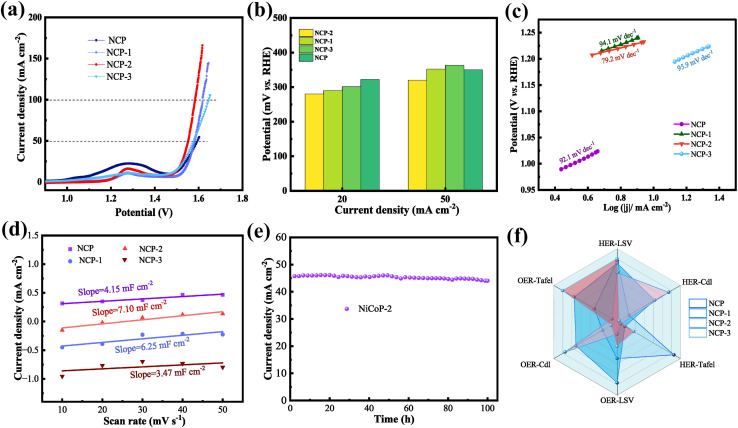
OER performance of the as-fabricated samples (a) LSV curves (b) overpotential values at different current density (c) Tafel curves (d) double-layer capacitance (*C*_dl_) (e) chronopotentiometric test (f) Radar image.

OER performances of the prepared materials are also investigated. Fig. 5a reveals the NCP-2 sample achieves overpotentials of 280, 320, 340 and 380 mV at current densities of 20, 50, 100 and 150 mA cm^−2^, respectively. It can still operate stably even at a high current density.

Fig. 5b presents a comparison of the overpotentials of NCP-2 and the other samples. At a current density of 20 mA cm^−2^, the overpotential of NCP-2 is lower than that of NCP (290.3 mV), NCP-1 (301.2 mV), NCP-3 (322.3 mV). At a current density of 50 mA cm^−2^, the overpotential of NCP-2 is still superior to that of the other samples.Table 2 shows that the overpotential of NCP-2 outperforms some of the previously reported data at a current density of 100 mA cm^−2^.^37–40^ Vanadium shows a strong electron-accepting ability, and it can optimize the catalytic performance of NiCoP by altering its electronic structure. When vanadium is incorporated into NiCoP, the electronic structure of vanadium may interact with the electronic structures of Ni and Co, thereby affecting the electron density distribution of the catalyst. This change can facilitate the adsorption and desorption of oxygen molecules, thereby lowering the energy barrier for the OER reaction and, in turn, reducing the overpotential. Meanwhile, as a transition metal, vanadium can alter the redox behavior on the catalyst surface. The incorporation of vanadium may help optimize the surface oxidation state transitions of Ni and Co, making it easier for NiCoP to form intermediate species with lower activation energies (such as *O, *OOH, *etc.*) during the catalytic process. The formation of these intermediate species helps reduce the overpotential for OER.

**Table 2:** A comparison for OER with previous reported Ni, Co-based material

Electrocatalysts	Overpotential (100 mA cm^−2)^	Electrolyte	References
MoP/Ni_2_P/NF	380 mV	1 M KOH	37
FeCo/Co_2_P@NPCF	452 mV	1 M KOH	38
CoP NFs	423 mV	1 M KOH	39
Co_2_P/CoNPC	471 mV	1 M KOH	40
NiCoP-2	340 mV	1 M KOH	This work

From Fig. 5c, NCP-2 exhibits the lowest Tafel slope of 79.2 mV dec^−1^, outperforming NCP (93.6 mV dec^−1^), NCP-1 (96.8 mV dec^−1^), NCP-3 (98.6 mV dec^−1^). This means that an appropriate amount of vanadium doping helps accelerate the kinetic reaction rate. This is because the appropriate introduction of vanadium may improve the interaction between the catalyst and reaction intermediates (such as *O, *OOH, *etc.*), leading to a faster OER reaction rate. The oxidation state transitions of Ni and Co on the NiCoP surface may occur more smoothly, thus accelerating the OER reaction. Additionally, the incorporation of vanadium may result in the formation of more active sites on the NiCoP surface, or optimize the electronic state of existing active sites, making it easier for reaction intermediates to react with the catalyst surface and reducing the rate-limiting steps of the reaction. These factors could all contribute to the decrease in the Tafel slope.

NCP-2 exhibits a *C*_dl_ of 7.10 mF cm^−2^, the highest among all the samples (Fig. 5d). It implies that the appropriate incorporation of vanadium can facilitate greater electron accumulation at the electrode surface, enhancing its charge transfer capabilities. From Fig. 5e, the NCP-2 sample still maintains excellent stability after 100 h of extended cycling. This sustained stability can be attributed to the robust structural integrity and high durability of NCP-2 under prolonged electrochemical stress, which allows it to effectively withstand the repeated charging and discharging cycles without significant loss of catalytic performance. The radar chart in Fig. 5f clearly demonstrates that the dual-functional electrocatalytic performance of NCP-2 surpasses that of the other five materials. The enhanced performance could stem from its optimal balance between conductivity, stability, and surface area, which allows it to facilitate multiple reaction pathways simultaneously. Such a multifaceted performance further underscores the potential of NCP-2 in practical applications.

After the OER cycling, the sample is further characterized by XRD, TEM, and XPS to elucidate the OER mechanism. As shown in Fig. S3, the diffraction peaks located at 37.5°, 43.4°, 54.2°, and 58.2° can be indexed to the (011), (210), (211), and (220) crystal planes of NiOOH (JPCDS no. 27-0956), respectively. Meanwhile, the peaks at 47.9°, 53.9°, 61.2°, and 63.0° are consistent with the (131), (211), (231), and (151) planes of CoOOH (JPCDS no. 26-0480), respectively. Two new phases are formed. The HRTEM images further illustrate this point in Fig. S4. The lattice spacing of 1.901 nm corresponds to the (131) crystal plane of CoOOH, while 1.987 nm corresponds to the (210) crystal plane of NiOOH, confirming that new CoOOH and NiOOH phases are indeed formed on the sample surface. This is because the V-doped NiCoP surface is no longer thermodynamically stable. OH^−^ nucleophilically attacks the P sites, oxidizing P to soluble phosphate and leaving behind P-vacancies. Simultaneously, the V dopant (V^3+^/V^4+^) acts as an electron pump, facilitating the oxidation of Ni^2+^ to Ni^3+^ and Co^2+^ to Co^3+^ by lowering the associated energy barriers. The resulting high-valent Ni^3+^ and Co^3+^ ions immediately coordinate with OH^−^ in the alkaline electrolyte, forming NiOOH and CoOOH phases. These oxyhydroxides are the true active layers for water oxidation.

Relative to the pre-cycling XPS spectrum, the Ni^2+^ contribution decreases, the Ni^3+^ fraction increases, and the satellite peak at 863 eV gains intensity (Fig. S5a). This evolution aligns with the preceding XRD and HRTEM findings, corroborating that the NiOOH phase has indeed formed. Likewise, the Co 2p peak shifts to lower binding energy, the Co^2+^ content decreases while Co^3+^ increases, all evidencing the formation of CoOOH (Fig. S5b). From Fig. S5c, the characteristic P 2p_3/2_ and P 2p_1/2_ peaks associated with metal–phosphide species nearly disappear, indicating the depletion of P^3−^ due to oxidative transformation. Meanwhile, the P–O peak becomes significantly broadened, suggesting the formation of multiple oxidized phosphorus species with diverse coordination environments. The V 2p_3/2_ peak show an obvious positive shift from 516.3 eV to 517.4 eV after OER cycling, accompanied by a significant increase in the V^5+^ species and a decrease in low-valence V^3+^/V^4+^ components, indicating the oxidation of vanadium during electrochemical activation. This suggests that the surface undergoes *in situ* reconstruction to form V-incorporated Ni (Co) oxyhydroxide as the real active phase for OER (Fig. S5d).

### 3.4 Overall water splitting measurement

**Fig. 6:**
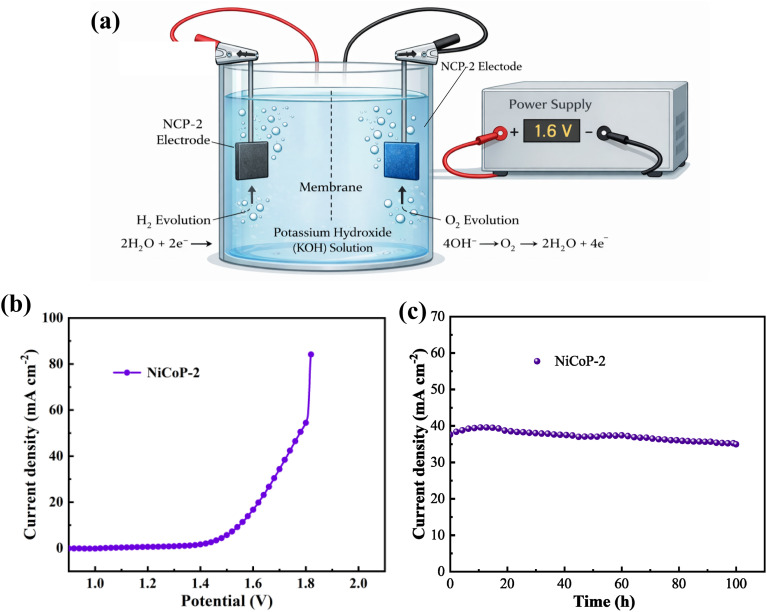
(a) Schematic illustration of overall water splitting (b) polarization curves at a scan rate of 5 mV s^−1^ (c) chronopotentiometry measurements.

Overall water splitting measurement is conducted using a 1M KOH electrolyte solution with NCP-2 sample as both the anode and cathode in a two-electrode system. The corresponding schematic diagram is shown in Fig. 6a. From the LSV curve in Fig. 6b, it can be seen that the NCP-2 sample owns a low cell voltage of 1.55 V at a current density of 10 mA cm^−2^, indicating that the material demonstrates a certain degree of superior catalytic performance in water electrolysis. The long-term cycling stability is also tested in Fig. 6c. It can be observed that there is no significant reduction in current density even at high current densities after 100 h. The good water splitting performance may be attributed to the following factors: first, the sample is directly grown on the foam nickel substrate, which not only provides more active sites but also helps improve the overall electrical conductivity of the material. Second, vanadium doping may alter the electronic structure of the NiCoP material, enhancing its catalytic activity in the water splitting reaction. Third, NiCoP materials possess good chemical stability, allowing them to maintain catalytic activity over long electrolysis periods and preventing material degradation, while also exhibiting excellent catalytic performance.

## 4. Conclusions

In summary, a binder-free electrode composed of V–NiCoP nanowires with controllable iron content is successfully fabricated on nickel foam *via* a hydrothermal method followed by phosphorization under an argon atmosphere. The as-prepared V-NiCoP exhibits excellent bifunctional electrocatalytic performance in 1.0 M KOH, delivering superior catalytic activity toward both the HER and OER compared with NiCoP. The NCP-2 sample shows an overpotential of 74.3 mV at 10 mA cm^−2^ with a small Tafel slope of 83.8 mV dec^−1^ during HER activity and 280 mV at 20 mA cm^−2^ (79.2 mV dec^−1^) for OER. Meanwhile, the NCP-2 nanowire arrays possess a low cell voltage of 1.55 V at 10 mA cm^−2^. Planar defects observed by HRTEM and XPS might increase the availability of active sites after V doping. The optimized adsorption energy of reaction intermediates on catalysts with suitable V content contributes to their selective HER and OER performance. Moreover, one-dimensional nanowire structures enable rapid electron transport along the longitudinal axis and expose abundant active sites with high surface-to-volume ratios, significantly enhancing the intrinsic catalytic activity for both HER and OER. The unique geometric morphology also facilitates efficient bubble detachment to prevent active site blocking and provides superior structural stability against aggregation during long-term electrocatalytic operations. This work could offer insights into the development of high-performance multimetallic phosphide bifunctional electrocatalysts for water splitting.

## Author contributions

Yongli Tong: writing – original draft; funding acquisition; Xuan Zhao: data curation; visualization; Yu Dong: validation; data curation; Ende Wang: supervision; project administration.

## Conflicts of interest

The authors declare no conflict of interest.

## Supplementary Material

RA-016-D5RA09888B-s001

## Data Availability

The data that support the findings of this study are available from the corresponding author upon reasonable request. Supplementary information (SI) is available. See DOI: https://doi.org/10.1039/d5ra09888b.
