# Supplementary material for: Bifunctional V-doped NiCoP nanowires for high-efficiency electrolysis
Source: RSC Adv. 2026 Mar 17;16(16):14555–65. doi: 10.1039/d5ra09888b (PMC12993930; doi:10.1039/d5ra09888b)
Supplement: RA-016-D5RA09888B-s001 [file RA-016-D5RA09888B-s001.pdf]

Supplementary Information (SI) for RSC Advances.
This journal is © The Royal Society of Chemistry 2026

Bifuntional V-doped NiCoP Nanowires for High-Efficiency Electrolysis

Yongli Tong [1, *], Xuan Zhao[1], Yu Dong[1], Ende Wang[2, *],

[1]School of Science, Shenyang Ligong University, Shenyang 110159, China;

[2]School of Materials Science and Engineering, Shenyang University of Technology, Shenyang 110870, China;

*Correspondence: tyl.tongyongli@163.com (Y. T.); ende_wang@163.com (E. Wang);

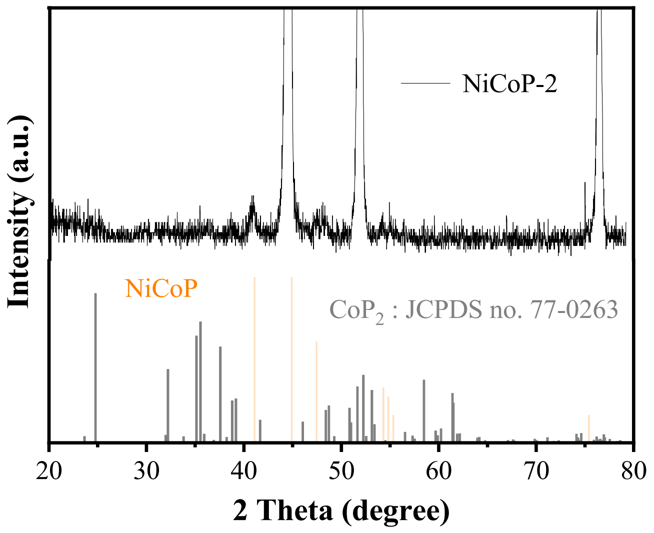

Figure S1 XRD image of the NiCoP-2 sample

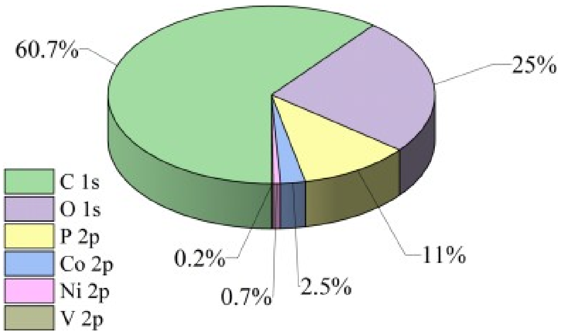

Figure S2 Elemental distribution percentage map

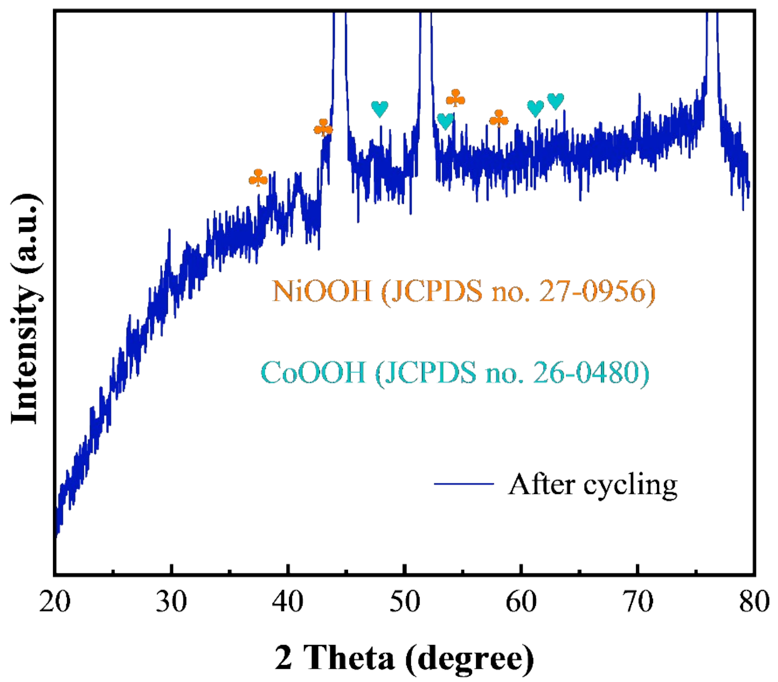

Figure S3 XRD of NiCoP-2 after cycling

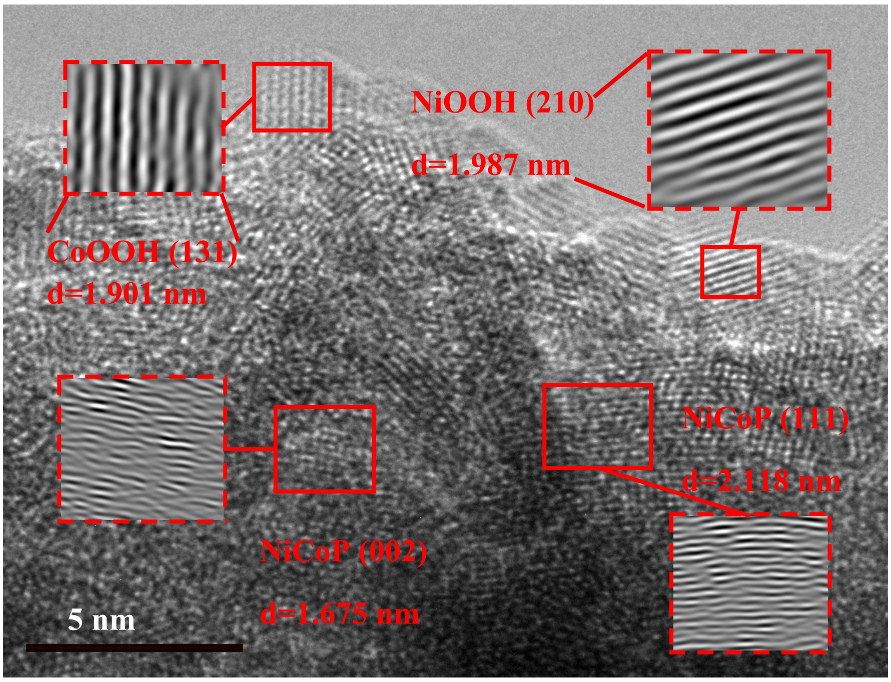

Figure S4 HRTEM of NCP-2 after cycling

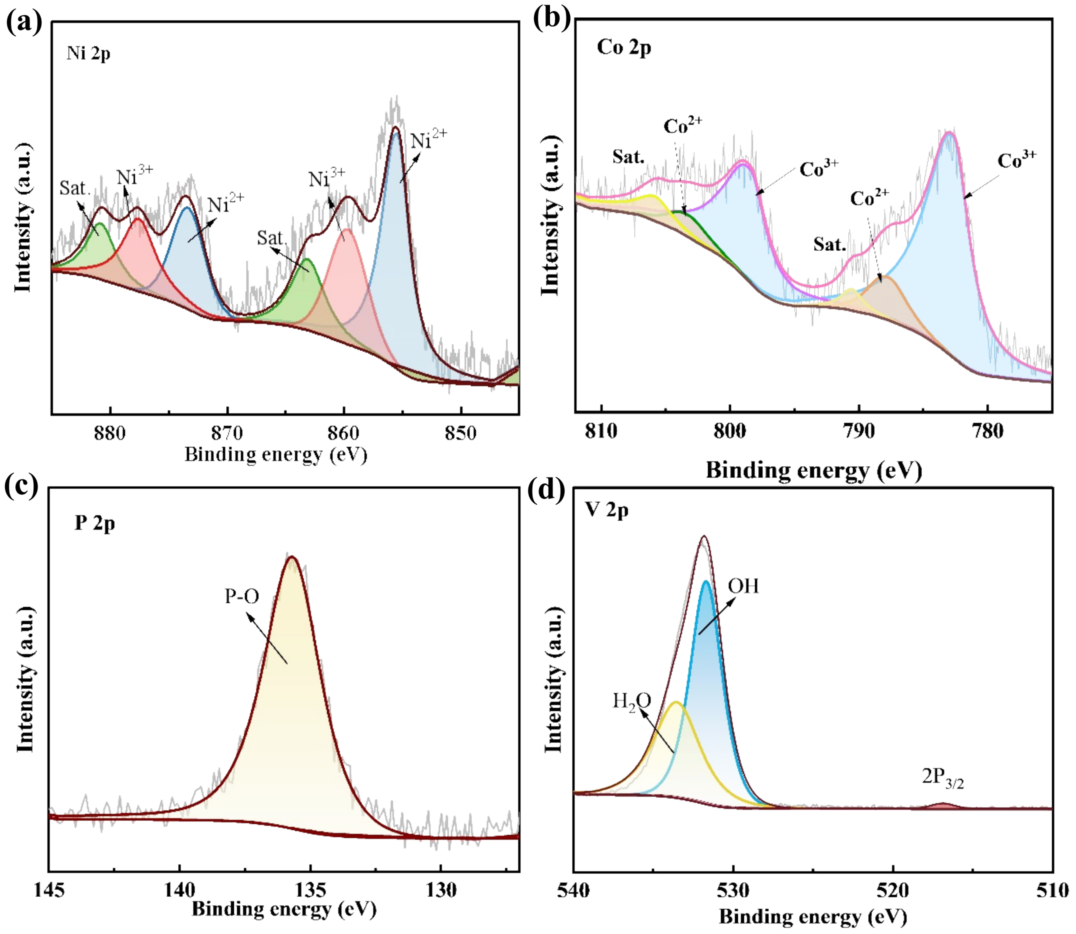

Figure S5 XPS of NCP-2 after cycling (a) Ni 2p (b) Co 2p (c) P 2p (d) V 2p
